# Multimodal imaging analysis of autosomal recessive bestrophinopathy: Case series

**DOI:** 10.1097/MD.0000000000038853

**Published:** 2024-07-19

**Authors:** Masahiro Miura, Shuichi Makita, Yoshiaki Yasuno, Shinnosuke Azuma, Toshihiro Mino, Takaaki Hayashi, Shuhei Kameya, Kazushige Tsunoda

**Affiliations:** a Department of Ophthalmology, Tokyo Medical University, Ibaraki Medical Center, Inashiki, Ibaraki, Japan; b Computational Optics Group, University of Tsukuba, Tsukuba, Ibaraki, Japan; c Topcon Corporation, Itabashi, Tokyo, Japan; d Department of Ophthalmology, The Jikei University School of Medicine, Minato, Tokyo, Japan; e Kameya Eye Clinic, Inzai, Chiba, Japan; f Division of Vision Research, National Institute of Sensory Organs, NHO Tokyo Medical Center, Meguro, Tokyo, Japan.

**Keywords:** autosomal recessive bestrophinopathy, case series, melanin, near-infrared autofluorescence, polarization-sensitive optical coherence tomography, retinal pigment epithelium

## Abstract

**Rationale:**

Autosomal recessive bestrophinopathy (ARB) is a subtype of bestrophinopathy caused by biallelic mutations of the *BEST1* gene, which affect the retinal pigment epithelium (RPE). Studying RPE abnormalities through imaging is essential for understanding ARB. This case series involved the use of multimodal imaging techniques, namely autofluorescence (AF) imaging at 488 nm [short-wavelength AF] and 785 nm [near-infrared AF (NIR-AF)] and polarization-sensitive optical coherence tomography (PS-OCT), to investigate RPE changes in 2 siblings with ARB.

**Patient concerns:**

Two Japanese siblings (Case 1: male, followed for 20–23 years; Case 2: female, followed for 13–17 years) carried compound heterozygous mutations of the *BEST1* gene.

**Diagnosis:**

Both siblings were diagnosed with ARB.

**Interventions and outcomes:**

Multimodal imaging techniques were used to evaluate RPE changes. Both siblings had funduscopic changes similar to those seen in the vitelliruptive stage of Best vitelliform macular dystrophy during the follow-up period. NIR-AF imaging showed hypo-AF of the entire macular lesion in both cases, and this hypo-AF remained stable over time. PS-OCT confirmed reduced RPE melanin content in these hypo-AF areas. Additionally, hyper-NIR-AF dots were observed within hypo-NIR-AF areas. Concomitant identification of focally thickened RPE melanin on PS-OCT imaging and hyper-AF on short-wavelength AF imaging at the sites containing hyper-NIR-AF dots indicated that the hyper-NIR-AF dots had originated from either stacked RPE cells or RPE dysmorphia.

**Lessons:**

We confirmed RPE abnormalities in ARB, including diffuse RPE melanin damage in the macula alongside evidence of RPE activity-related changes. This case series demonstrates that multimodal imaging, particularly NIR-AF and PS-OCT, provides detailed insights into RPE alterations in ARB.

## 1. Introduction

Bestrophinopathy encompasses a spectrum of inherited macular degenerations caused by mutations in the *BEST1* gene.^[1]^ Mutations in the *BEST1* gene can result in abnormal functioning of the protein bestrophin-1, which is believed to function as an anion channel in the retinal pigment epithelium (RPE). Thus, abnormal functioning of this protein leads to a variety of retinopathies.^[1]^ Autosomal recessive bestrophinopathy (ARB) (OMIM-611809) is a subtype of bestrophinopathy caused by biallelic mutations of the *BEST1* gene.^[2]^ Clinical findings in patients with ARB differ from those in patients with Best vitelliform macular dystrophy (BVMD). ARB is associated with various fundus abnormalities, including subretinal fluid, intraretinal fluid, multiple yellow-white flecks, multiple RPE atrophies, and scar lesions.^[2–6]^ Vitelliform lesions, which are typical for BVMD, are not observed in ARB.^[2,3,5,6]^

Considering that the *BEST1* gene is associated with the RPE, the abnormalities in patients with ARB are thought to originate from RPE dysfunction. Therefore, analyzing RPE abnormalities through imaging is crucial for understanding the pathology of ARB. Clinical autofluorescence (AF) imaging is a widely used technique for evaluating RPE changes in retinal diseases. In healthy eyes, short-wavelength AF (SW-AF) (excitation of 488 nm) signals predominantly originate from lipofuscin or melanolipofuscin in RPE cells,^[7]^ while near-infrared AF (NIR-AF) (excitation of 785 nm) signals primarily arise from melanin or melanolipofuscin in RPE cells.^[8]^ In ARB-affected eyes, SW-AF imaging often reveals hyper-AF lesions corresponding to the observed yellow-white flecks.^[2,3,5,6]^ Conversely, NIR-AF imaging typically shows a broad hypo-AF area in the macula.^[6]^ While RPE dysfunction is a potential source of this hypo-AF area, absorption of emitted light by intraretinal or subretinal fluid is another possibility.^[8]^ One drawback of AF imaging is its inability to provide topographical information, which can hinder the interpretation of hypo-NIR-AF lesions.

Intensity-based optical coherence tomography (OCT) imaging (so-called “standard” OCT imaging) is another crucial tool for assessing RPE changes. In age-related macular degeneration (AMD), for example, RPE damage manifests as a zone of attenuation or disruption in the RPE–Bruch membrane band accompanied by hyper-transmission to the choroid.^[9]^ While standard OCT offers valuable insights into RPE alterations, its ability to evaluate RPE cells directly is limited because of the lack of specific RPE contrast, making direct comparisons with AF images challenging. Polarization-sensitive OCT (PS-OCT), a functional extension of OCT technology, enables enhanced tissue differentiation in retinal diseases.^[10]^ The multiple light scattering caused by melanin in the RPE induces depolarization, which PS-OCT can detect.^[11]^ Combining PS-OCT with AF imaging facilitates comprehensive three-dimensional evaluation of RPE changes.^[12]^

In this case series, we employed multimodal imaging (AF and PS-OCT) to investigate RPE alterations in 2 siblings with ARB.

## 2. PS-OCT imaging

A detailed description of the prototype PS-OCT system has been previously reported.^[13,14]^ The PS-OCT system is a multifunctional swept-source OCT device with a 1-μm wavelength band and has a polarization-diversity detection capability. This multifunctional OCT device provides standard OCT images and degree of polarization uniformity (DOPU)^[15]^ images from a single measurement. The DOPU was calculated with Makita noise correction using a 3- × 3-pixel kernel (35 μm and 13 μm along the lateral and axial directions, respectively).^[16]^ The presence of a low DOPU indicated depolarization by the melanin-induced scattering of multiple lights.^[15]^ To specify the location of the depolarization observed on the standard OCT image, composite DOPU B-scan OCT images were created. In these images, the area of low DOPU (<0.8) was overlaid on the standard OCT B-scan image with red color. A raster scanning protocol with 512 A-lines × 256 B-scans covering a 6.0- × 6.0-mm region of the retina was used for volumetric scans. OCT volumes without significant motion artifacts were used for this study.

To estimate the thickness of the RPE melanin layer, we calculated the thickness of the region with low DOPU in the retina. First, to distinguish RPE melanin from choroidal melanin, the outer border of the RPE–Bruch membrane complex was automatically segmented using a method identical to the built-in algorithm of a commercially available OCT device (DRI-OCT Triton; Topcon, Tokyo, Japan). Second, pixels with low DOPU (<0.8) were identified within the inner retinal layer from the segmented line. Third, the number of low-DOPU pixels was summed along each A-scan within the three-dimensional PS-OCT dataset. The resulting two-dimensional *en face* projection map, called the RPE melanin thickness map, depicted the distribution of depolarizing material, primarily representing RPE melanin, within the retina.

## 3. Multimodal imaging

We conducted a multimodal image analysis using color fundus photographs (fundus camera combined with 3D OCT 2000 or DRI-OCT Triton; Topcon) and commercial OCT (3D OCT 2000 or DRI-OCT Triton; Topcon). For Case 1, the DRI-OCT Triton was used throughout the follow-up period. For Case 2, the 3D OCT 2000 was used at the age of 13 and 15 years and the DRI-OCT Triton was used at the age of 17 years. Both NIR-AF images and SW-AF images were obtained using HRA2 (Heidelberg Engineering, Heidelberg, Germany).

## 4. Ethics statement

The present study adhered to the tenets of the Declaration of Helsinki and was approved by the Institutional Review Board of Tokyo Medical University (approval number: IRB-861, T2019-0072). The nature of the study and the implications of participation were explained to all participants. Written informed consent for the publication of this case report, including the accompanying images, was obtained from the patients and their parents after a detailed explanation of the report was provided and their full understanding was ensured.

## 5. Cases

Proband 1 (Case 1) and proband 2 (Case 2) were Japanese siblings. Case 1 was a male patient who was followed up from 20 to 23 years of age. Case 2 was his younger sister by 2 years, and she was followed up from 13 to 17 years of age. Both patients presented with visual impairments. However, their best-corrected visual acuity remained 1.0 in both eyes throughout the follow-up period.

To identify mutations of the causative genes, genomic DNA was extracted from peripheral blood samples of the two Japanese siblings, designated Cases 1 and 2 as noted above. DNA isolation for Case 1 was performed using a Gentra Puregene Blood Kit (Qiagen, Hilden, Germany), and that for Case 2 was performed using a NucleoSpin Blood XL (Macherey–Nagel, Düren, Germany). Whole-exome sequencing was conducted as described in previous reports.^[17–20]^ The analysis, following a series of filtration steps, revealed identical compound heterozygous mutations (c.404>T, p.Ala135Val and c.583>T, p.Arg195Trp) in the *BEST1* gene in both patients.

### 5.1. Case 1

**Figure 1.**
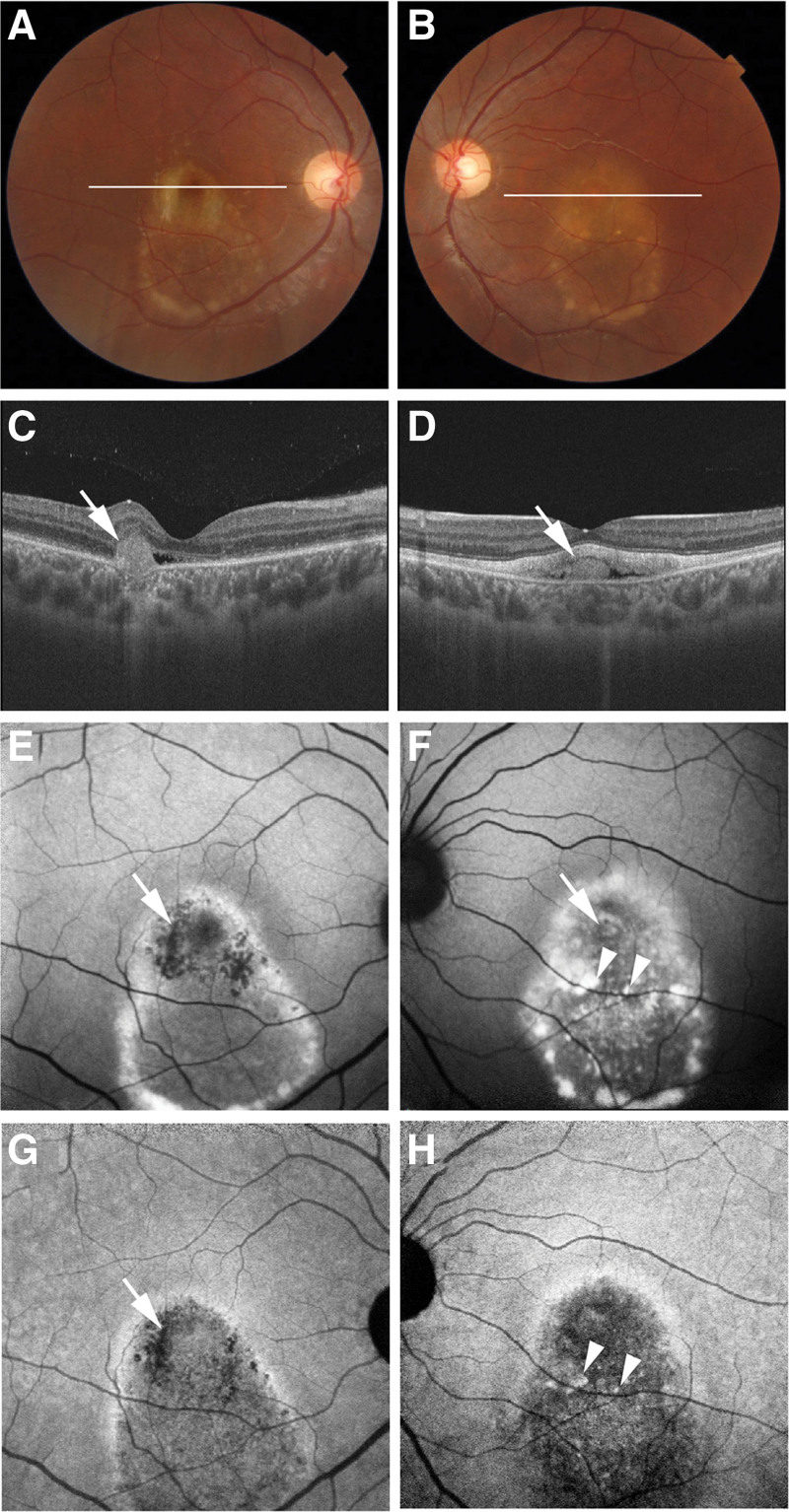
Multimodal imaging of Case 1 at the age of 20 years. (A and B) Color fundus photographs of both eyes showed findings similar to those of the vitelliruptive stage. White lines in the (A) right and (B) left eyes indicated the scanning lines of commercial optical coherence tomography (OCT) images of the (C) right and (D) left eyes, respectively. (C) The white arrow in the OCT image of the right eye indicates subretinal hyper-reflective material. (D) The white arrow in the OCT image of the left eye indicates hyper-reflective material in the subretinal fluid. (E) The white arrow in the short-wave autofluorescence (AF) image of the right eye indicates hypo-AF at the subretinal hyper-reflective material. (F) The white arrow in the short-wave AF image of the left eye indicates hyper-AF at the hyper-reflective material. (G) The white arrow in near-infrared AF image of the right eye indicates hypo-AF at the subretinal hyper-reflective material. (H) The arrowheads in the near-infrared AF images of the left eye indicate hyper-AF dots. (F) The short-wave AF image also showed hyper-AF dots at corresponding locations (arrowheads). (G, H) The near-infrared AF images of both eyes showed hypo-AF of the entire macular lesions.

**Figure 2.**
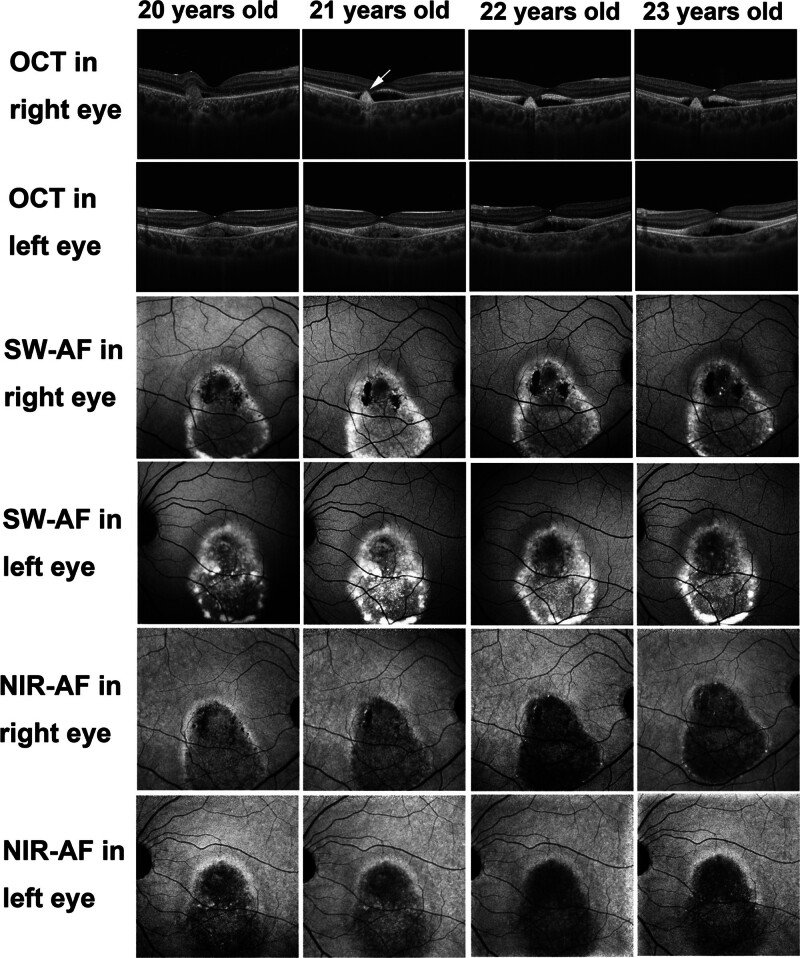
Multimodal imaging of Case 1 during the follow-up period. Commercial optical coherence tomography (OCT) imaging revealed subretinal fluid in both eyes throughout the follow-up period. The OCT image of the right eye showed increased reflectivity and a distinct border in the subretinal hyper-reflective material at the age of 21 years (white arrow). The hyper-reflective material in the left eye had diminished on OCT imaging by the age of 22 years. The hyper-short-wave autofluorescence area in both eyes changed over time. The hypo-near-infrared autofluorescence area remained unchanged throughout the follow-up period. NIR-AF = near-infrared autofluorescence; OCT = optical coherence tomography; SW-AF = short-wave autofluorescence.

The funduscopic changes throughout the follow-up period resembled those in eyes with BVMD at the vitelliruptive or scrambled-egg stage (Fig. 1A and B). Initial commercial OCT of the right eye at the age of 20 years revealed subretinal fluid and subretinal hyper-reflective material (Fig. 1C). Hyper-transmission to the choroid confirmed focal RPE damage underlying the hyper-reflective material. At 21 years of age, follow-up revealed increased reflectivity and a distinct border in the subretinal hyper-reflective material (Fig. 2). The left eye exhibited subretinal fluid containing hyper-reflective material (Fig. 1D). Elongated photoreceptor outer segments were also observed in the left eye. By the age of 22 years, the hyper-reflective material in the left eye had diminished (Fig. 2).

SW-AF imaging at the age of 20 years showed hyper-AF areas corresponding to yellow-white lesions on color fundus photographs of both eyes (Fig. 1E and F). The hyper-reflective material in the left eye also showed a hyper-AF area (Fig. 1F). The hyper-SW-AF area in both eyes changed over time (Fig. 2). Additionally, the right eye displayed a hypo-SW-AF area corresponding to the focal RPE damage beneath the subretinal hyper-reflective material (Fig. 1E). NIR-AF imaging revealed hypo-AF in the entire lesion area of both eyes (Fig. 1G and H), and this hypo-AF remained unchanged throughout the follow-up period (Fig. 2). Notably, SW-AF imaging (Fig. 1E and F) failed to clearly depict the previously identified hypo-AF in the entire lesion. NIR-AF imaging of the right eye (Fig. 1G) showed a hypo-AF area corresponding to the focal RPE damage. Hyper-NIR-AF dots were observed in the left eye (Fig. 1H), and the number of hyper-NIR-AF dots decreased as the follow-up period progressed (Fig. 2). These hyper-AF dots on NIR-AF imaging also appeared hyper-AF on SW-AF imaging (Fig. 1F).

**Figure 3.**
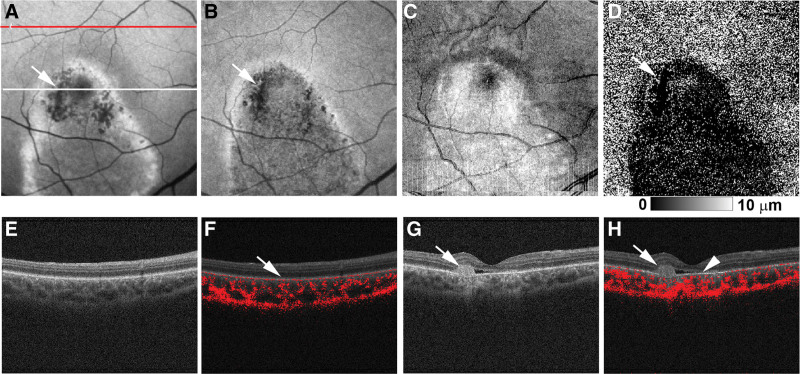
Polarization-sensitive optical coherence tomography (OCT) imaging of the right eye of Case 1 at the age of 20 years. (A) The short-wave autofluorescence (AF) image shows the scanning lines of B-scan OCT imaging in (E, F) (red line) and (G, H) (white line). (B) The near-infrared AF image showed hypo-AF in the entire lesion. (C) Projection image of standard OCT. (D) The retinal pigment epithelium (RPE) melanin thickness map showed a reduction of RPE melanin in the entire lesion. (E) Standard OCT image outside the macular lesion. (F) The composite DOPU B-scan OCT image outside the macular lesion showed depolarization by RPE melanin (white arrow). (G) The standard OCT image at the fovea showed subretinal hyper-reflective material (white arrow) with focal RPE damage. Both the (A) short-wave AF image and (B) near-infrared AF image showed hypo-AF at the site of focal RPE damage (white arrows). (D) In the RPE melanin thickness map, RPE melanin showed further decreases corresponding to the focal RPE damage (white arrow). (H) The composite DOPU B-scan OCT image showed the absence of depolarization at the subretinal hyper-reflective material (white arrow) and reduction of RPE melanin (white arrowhead).

**Figure 4.**
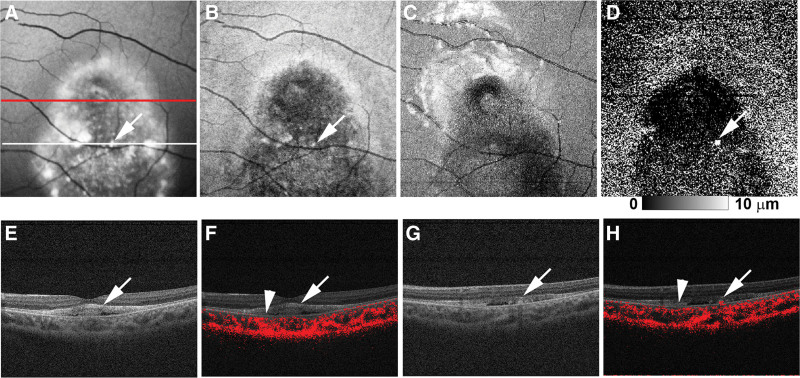
Polarization-sensitive optical coherence tomography (OCT) imaging of the left eye of Case 1 at the age of 20 years. (A) The short-wave autofluorescence (AF) image shows the scanning lines of B-scan OCT images in (E, F) (red line) and (G, H) (white line). The white arrows in the (A) short-wave AF image and (B) near-infrared AF image indicate hyper-AF dots. (C) Projection image of standard OCT. (D) The retinal pigment epithelium (RPE) melanin map showed reduction of RPE melanin in the entire lesion. The RPE melanin showed increases corresponding to hyper-AF dots (white arrow). (E) The standard OCT image at the fovea showed hyper-reflective material in the subretinal fluid (white arrow). (F) The composite DOPU B-scan OCT image showed the absence of depolarization at the hyper-reflective material (white arrow) and a reduction of RPE melanin (white arrowhead). (G) The standard OCT image below the macula showed focal thickening of the RPE–Bruch membrane band (white arrow) at the (A, B) hyper-AF dots. (H) The composite DOPU B-scan OCT image showed accumulation of RPE melanin (white arrow) and reduction of RPE melanin (white arrowhead).

PS-OCT measurement conducted at the age of 20 years (Figs. 3A–H and 4A–H) confirmed decreased RPE melanin thickness within the hypo-NIR-AF area of both eyes (Figs. 3D and 4D). In the right eye, this reduction was further pronounced in the area of focal RPE damage with hyper-transmission to the choroid (Fig. 3D). Composite DOPU B-scan OCT images demonstrated preserved RPE melanin outside the vitelliruptive lesion (Fig. 3F) and decreased melanin within it (Figs. 3H and 4F, H). Notably, both NIR-AF and PS-OCT imaging demonstrated a decrease in RPE melanin throughout the macular lesion. However, the integrity of the RPE–Bruch membrane band was preserved on standard OCT imaging, showing no attenuation, disruption, or hyper-transmission to the choroid (Figs. 3G and 4E, G). Neither the subretinal hyper-reflective material in the right eye nor the hyper-reflective material in the left eye exhibited depolarization on composite DOPU B-scan OCT images (Figs. 3H and 4F). Finally, standard OCT imaging in the left eye (Fig. 4G) revealed focal thickening of the RPE–Bruch membrane band at the hyper-AF dots (Fig. 4A and B). Composite DOPU B-scan images depicted melanin accumulation at the site of this lesion (Fig. 4H).

### 5.2. Case 2

**Figure 5.**
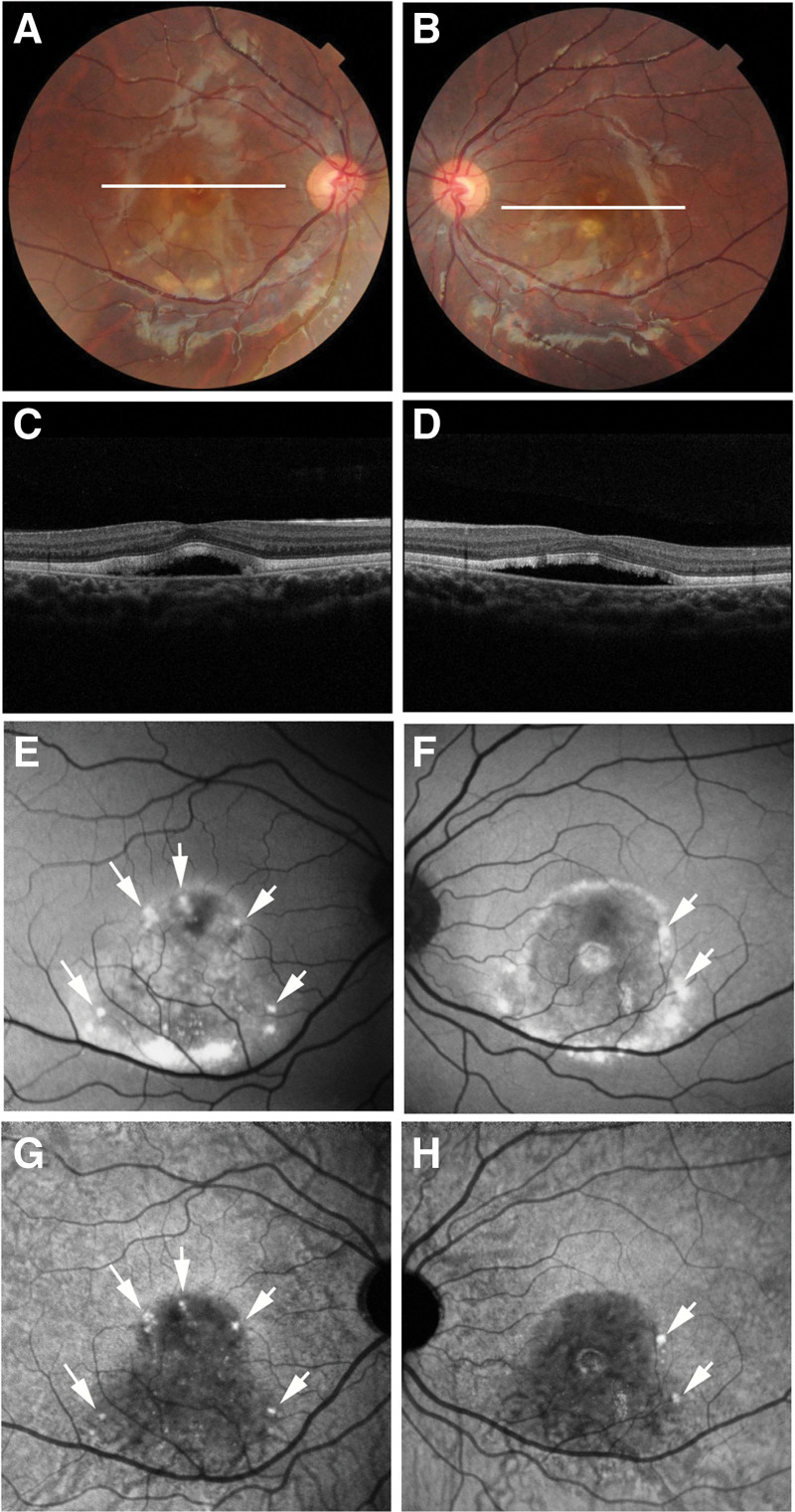
Multimodal imaging of Case 2 at the age of 13 years. (A, B) The findings in the color fundus photographs of both eyes were similar to those in the vitelliruptive stage. The white lines in the (A) right and (B) left eyes indicate the scanning lines of the commercial optical coherence tomography (OCT) images of the (C) right and (D) left eyes, respectively. (C, D) The OCT images of both eyes showed subretinal fluid. The white arrows in the short-wave autofluorescence (AF) images of the (E) right and (F) left eyes indicate hyper-AF dots corresponding to the hyper-AF dots in the near-infrared AF images of the (G) right and (H) left eyes, respectively.

**Figure 6.**
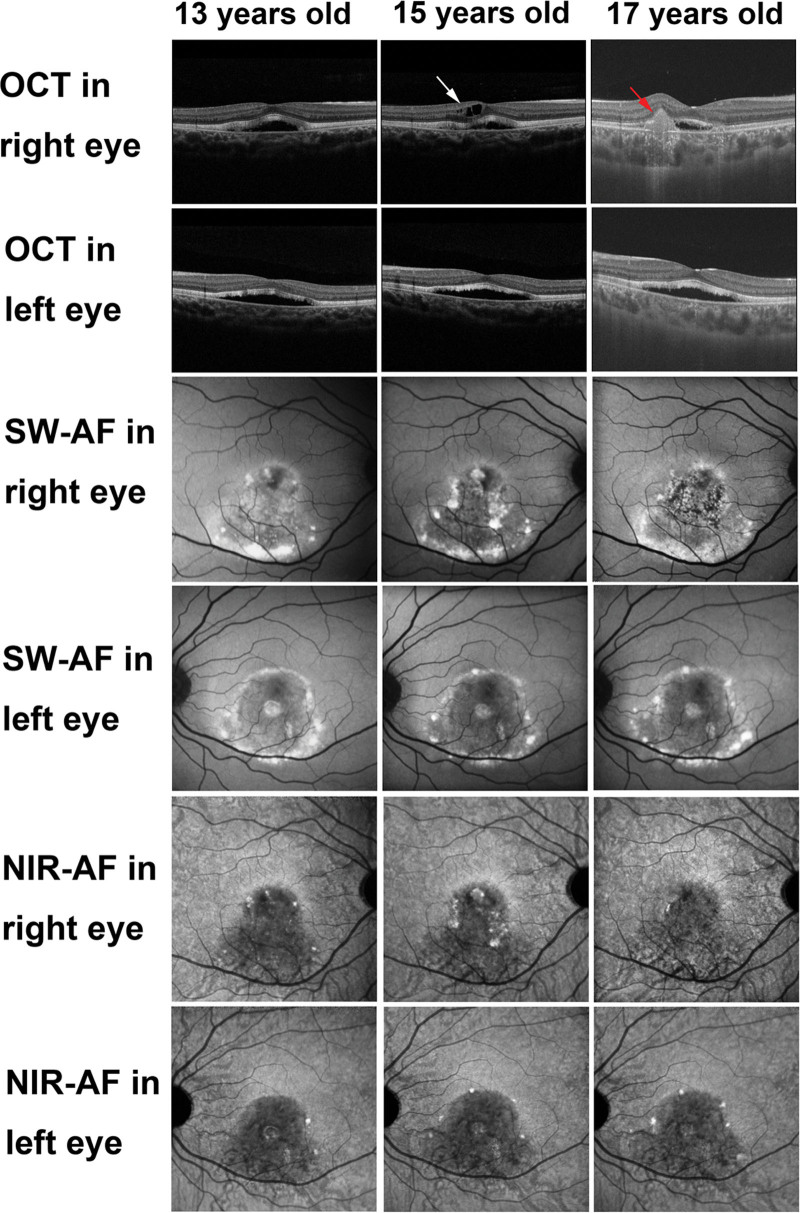
Multimodal imaging of Case 2 during the follow-up period. Commercial optical coherence tomography imaging revealed subretinal fluid in both eyes throughout the follow-up period. The right eye additionally showed intraretinal fluid at the age of 15 years (white arrow) and development of subretinal hyper-reflective material at the age of 17 years (red arrow). The hyper-short-wave autofluorescence (AF) area in both eyes changed over time. The hypo-near-infrared-AF area remained unchanged throughout the follow-up period. The number of hyper-near-infrared-AF dots increased in both eyes throughout the follow-up period. NIR-AF = near-infrared autofluorescence; OCT = optical coherence tomography; SW-AF = short-wave autofluorescence.

The funduscopic findings throughout the follow-up period remained consistent with the vitelliruptive stage of BVMD (Fig. 5A and B). Initial commercial OCT at the age of 13 years revealed subretinal fluid with elongated photoreceptor outer segments in both eyes (Fig. 5C and D). This subretinal fluid persisted throughout the follow-up period (Fig. 6). At the age of 15 years, intraretinal fluid was observed in the right eye (Fig. 6). At the age of 17 years, the right eye developed subretinal hyper-reflective material accompanied by focal RPE damage confirmed by hyper-transmission to the choroid (Fig. 6).

SW-AF imaging (Fig. 5E and F) identified a hyper-AF area corresponding to the yellow-white lesions seen on color fundus photographs. This hyper-AF area changed in size over time (Fig. 6). Interestingly, the right eye also exhibited a mottled hypo-AF appearance at the age of 17 years (Fig. 6). NIR-AF imaging revealed hypo-AF in the entire macular lesion of both eyes (Fig. 5G and H), which remained unchanged throughout the follow-up period (Fig. 6). However, SW-AF imaging (Fig. 5E and F) was less successful in capturing this entire hypo-AF region. Within the hypo-NIR-AF areas, hyper-NIR-AF dots were observed (Fig. 5G and H), increasing in number in both eyes over the follow-up period (Fig. 6). These hyper-NIR-AF dots also exhibited a hyper-AF appearance on SW-AF imaging (Fig. 5E and F).

**Figure 7.**
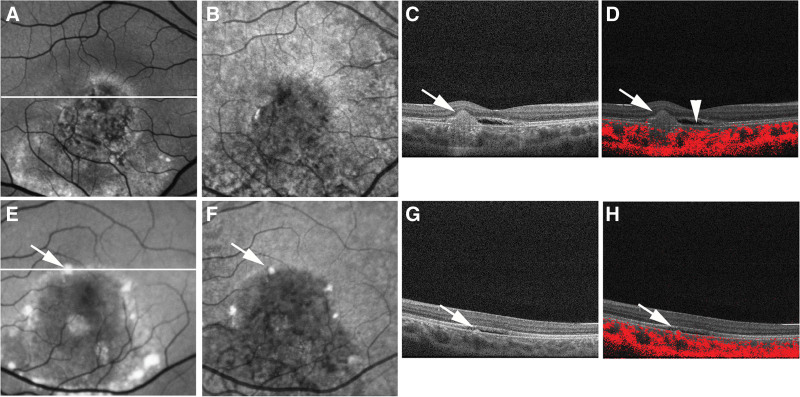
Polarization-sensitive optical coherence tomography (OCT) imaging of Case 2 at the age of 17 years. (A) The white line in the short-wave autofluorescence (AF) image of the right eye indicates the (C, D) scanning line of the B-scan OCT images in the right eye. (B) Near-infrared AF image of the right eye. (C) The standard OCT image of the right eye showed subretinal hyper-reflective material. (F) The composite DOPU B-scan OCT image showed the absence of depolarization at the hyper-reflective material (white arrow) and a reduction of RPE melanin (white arrowhead). (E) The white line in the short-wave autofluorescence (AF) image of the left eye indicates the (G, H) scanning line of the B-scan OCT images of the left eye. (F) Near-infrared AF image of the left eye. (G) The standard OCT image of the left eye showed focal thickening of the RPE–Bruch membrane band (white arrow) at the hyper-AF dots in the (E) short-wave AF image (white line) and (F) near-infrared AF image (white line). (H) The composite DOPU B-scan OCT image of the left eye showed accumulation of RPE melanin (white line).

PS-OCT measurement conducted at the age of 17 years (Fig. 7A–H) showed a decrease in RPE melanin within the hypo-NIR-AF area (Fig. 7D, H). Similar to Case 1, no attenuation or disruption was observed in the RPE–Bruch membrane band, and no hyper-transmission to the choroid was detected within the area of RPE melanin reduction on standard OCT images (Fig. 7C, G). Importantly, the composite DOPU B-scan OCT images revealed no depolarization at the subretinal hyper-reflective material in the right eye (Fig. 7D). Finally, standard OCT imaging in the left eye (Fig. 7G) revealed focal thickening of the RPE–Bruch membrane band at the hyper-NIR-AF dots, with corresponding melanin accumulation visualized on the composite DOPU B-scan images (Fig. 7H).

## 6. Discussion

Previous reports have described both NIR-AF and PS-OCT findings in eyes with ARB.^[4,6]^ However, prior studies have independently utilized PS-OCT and NIR-AF to investigate RPE changes in ARB. The present case series uniquely presents a comprehensive comparison of these imaging modalities to gain deeper insights into ARB-related RPE alterations.

NIR-AF imaging revealed widespread hypo-AF within the macular lesions, suggesting RPE dysfunction. The PS-OCT findings corroborated this, demonstrating a decrease in RPE melanin content in the corresponding areas. However, the hypo-AF area on NIR-AF imaging was not apparent on SW-AF imaging in this case series, consistent with previous reports of NIR-AF in ARB.^[6]^ Similarly, other bestrophinopathies such as BVMD exhibit hypo-NIR-AF lesions not seen in SW-AF, even in subclinical stages.^[21]^ Despite the decrease in RPE melanin, no findings suggestive of RPE damage were observed on standard OCT images. Specifically, no attenuation or disruption was observed in the RPE–Bruch membrane band, and no hyper-transmission to the choroid was detected. This suggests selective melanin damage in RPE cells in eyes with ARB. Bestrophin-1, encoded by the *BEST1* gene, is a multifunctional protein in the RPE. It forms a calcium-activated chloride channel and regulates intracellular voltage-dependent calcium channels.^[22–24]^ Calcium influences electrical activity and melanin storage in melanophores, much like its role in regulating these processes in the RPE.^[25,26]^ Therefore, it is plausible that *BEST1* mutations in patients with ARB disrupt calcium homeostasis due to altered chloride conductance, impacting melanin pathways. Taken together, these findings suggest that hypo-AF on NIR-AF imaging in patients with ARB likely arises from selective RPE melanin damage.

NIR-AF imaging in this study revealed hyper-AF dots. Standard OCT showed focal thickening of the RPE–Bruch membrane band at these hyper-NIR-AF dots, hinting at potential structural changes. Further supporting this, PS-OCT confirmed increased melanin accumulation in these lesions, with SW-AF imaging concurrently detecting lipofuscin accumulation. Notably, the co-occurrence of lipofuscin and melanin suggests that the possible origin of the hyper-AF dots is stacked RPE cells or RPE dysmorphia.^[12]^ Both stacked RPE cells and RPE dysmorphia potentially reflect reactive changes to RPE dysfunction, similar to the findings in AMD.^[27]^ Interestingly, similar reactive changes in AMD, such as intraretinal RPE migration, have been reported in the eyes of patients with BVMD using PS-OCT.^[4]^ This suggests a potential commonality in RPE responses to dysfunction across different retinal diseases. Furthermore, the possible evolution of hyper-NIR-AF dots over time hints at parallels with AMD, wherein RPE activity can dynamically change. Further studies are needed to confirm these findings and explore the long-term course of RPE activity in ARB.

Cases 1 and 2 both showed subretinal hyper-reflective material on follow-up. The subretinal hyper-reflective material was hypo-reflective on both SW-AF and NIR-AF imaging and was associated with focal RPE damage. Additionally, no depolarization was observed on PS-OCT imaging. These findings suggest that the subretinal hyper-reflective material does not contain RPE, lipofuscin, or unphagocytosed photoreceptor outer segments. This finding may originate from fibrotic scar tissue, but further investigation is needed.

SW-AF imaging revealed hyper-AF signals corresponding to hyper-reflective material seen in the subretinal fluid on standard OCT images. Notably, PS-OCT confirmed the absence of melanin in this material, consistent with previous reports suggesting that hyper-SW-AF lesions in ARB arise from accumulations of lipofuscin and unphagocytosed photoreceptor outer segments.^[1,28]^ Furthermore, observations suggest that these accumulations might evolve over time, mirroring potential changes in RPE function in ARB. This underscores the need for long-term studies to fully elucidate the evolving clinical pathophysiology of ARB.

This case series has several limitations. First, ARB manifests with a diverse range of fundus abnormalities, and this study only examined a limited subset of these features. Second, the natural history of ARB remains incompletely understood because the disease process itself can exhibit dynamic changes over time. Addressing these limitations will require larger and longer-term studies. Third, although a prior study suggested a direct relationship between DOPU and melanin content,^[11]^ RPE melanin thickness measurements may be influenced by factors such as the shape and size of the spatial kernel to compute the DOPU^[29]^ as well as the melanin arrangement within RPE cells.^[15,30]^ Consequently, RPE melanin thickness maps, despite reflecting trends in RPE melanin content, do not provide an absolute measure of the actual thickness of RPE melanin.

In conclusion, we have herein described two siblings diagnosed with ARB. Multimodal imaging (the combination of PS-OCT and NIR-AF) allowed us to confirm diffuse RPE melanin damage in the macula alongside evidence of RPE activity-related changes. This highlights the valuable contribution of multimodal imaging, particularly NIR-AF and PS-OCT, in comprehensively evaluating RPE alterations in ARB.

## Acknowledgments

This study was supported by Grants-in-Aid for Scientific Research (21K09684 and 24K12753) from the Japan Society for the Promotion of Science. We thank Angela Morben, DVM, ELS, from Edanz (https://jp.edanz.com/ac) for editing a draft of this manuscript.

## Author contributions

**Conceptualization:** Masahiro Miura, Takaaki Hayashi, Shuhei Kameya, Kazushige Tsunoda.

**Data curation:** Masahiro Miura, Takaaki Hayashi, Shuhei Kameya, Kazushige Tsunoda.

**Formal analysis:** Masahiro Miura, Yoshiaki Yasuno, Toshihiro Mino, Takaaki Hayashi, Kazushige Tsunoda.

**Funding acquisition:** Masahiro Miura.

**Investigation:** Masahiro Miura, Shuichi Makita, Yoshiaki Yasuno, Shinnosuke Azuma, Toshihiro Mino, Takaaki Hayashi, Shuhei Kameya, Kazushige Tsunoda.

**Methodology:** Masahiro Miura, Shuichi Makita, Shinnosuke Azuma, Toshihiro Mino, Takaaki Hayashi.

**Project administration:** Masahiro Miura, Shuhei Kameya.

**Resources:** Masahiro Miura, Shuichi Makita, Yoshiaki Yasuno, Shinnosuke Azuma, Takaaki Hayashi.

**Software:** Masahiro Miura, Shuichi Makita, Yoshiaki Yasuno, Shinnosuke Azuma, Toshihiro Mino.

**Supervision:** Masahiro Miura.

**Validation:** Masahiro Miura, Kazushige Tsunoda.

**Visualization:** Masahiro Miura, Shuichi Makita, Yoshiaki Yasuno, Toshihiro Mino.

**Writing – original draft:** Masahiro Miura.

**Writing – review & editing:** Masahiro Miura, Shuichi Makita, Yoshiaki Yasuno, Shinnosuke Azuma, Toshihiro Mino, Takaaki Hayashi, Shuhei Kameya, Kazushige Tsunoda.
